# Role of Prophylactic Oral Antibiotics in the Prevention of Post-cataract Surgery Acute Infective Endophthalmitis

**DOI:** 10.7759/cureus.42662

**Published:** 2023-07-29

**Authors:** Aruba Zafar, Fiza Shaheen, Tahira Afzal, Sabihuddin Ahmad, Muhammad Amjad

**Affiliations:** 1 Department of Ophthalmology, Al Shifa Trust Eye Hospital, Rawalpindi, PAK; 2 Department of Vitreoretina, Al Shifa Trust Eye Hospital, Rawalpindi, PAK

**Keywords:** infectious endophthalmitis, post-operative complications, phacoemulsification, cataract extraction, antibiotic prophylaxis

## Abstract

Purpose

To evaluate the role of perioperative oral antibiotics in the prevention of acute infective endophthalmitis (IE) after cataract surgery.

Methods

A prospective cohort study of patients older than 18 years of age undergoing uncomplicated phacoemulsification was conducted. Group A was given post-op oral ciprofloxacin for three days, whereas Group B was not. Both groups received 5% povidone-iodine (PVI) preparation for five minutes in the conjunctival cul-de-sac, and intracameral (IC) 0.5% moxifloxacin was administered at the end of the procedure as prophylaxis. The minimum post-op follow-up period was six weeks.

Results

Out of 2161 patients, 859 (39.8%) were included in Group A, and 1302 (60.2%) were included in Group B. No significant difference in anterior chamber (AC) reaction was found on day 1 (p = 0.67), day 14 (p = 0.03), or day 45 (p = 0.1). One patient developed acute post-op IE (0.04%) and two patients developed toxic anterior segment syndrome (TASS) from Group A. The non-oral antibiotic group had no serious complications.

Conclusion

Perioperative oral antibiotic use in routine clinical practice is not recommended for the prevention of acute post-op IE. Pre-op conjunctival PVI 5% for five minutes and IC moxifloxacin at the end of surgery were proven to be effective prophylactic measures in our study.

## Introduction

Cataracts are a major cause of global blindness, and cataract extraction is one of the most commonly performed surgical procedures in ophthalmic clinical settings. Exogenous infective endophthalmitis (IE) following cataract surgery, although rare, is a serious and sight-threatening complication that frequently leads to poor vision (usually less than 20/200) or even loss of the eye [1,2]. The incidence of IE after cataract surgery is low but varies substantially in the literature from 0.012% to 0.56%, with a large meta-analysis reporting it to be 0.134% (n = 6,686,169) on average [3-5].

Multiple factors can increase the risk of post-op IE [6]. Ocular pathologies like blepharitis, ectropion, conditions involving an increased number of ocular bacteria, and temporal and clear corneal incisions are some of the well-known risk factors [2]. Patients on immunosuppressants, older patients, and those with wound dehiscence are also at a higher risk for this condition [6].

Prophylaxis against post-op IE can be achieved with topical, intracameral (IC), or systemic antibiotics. Standard international recommendations mainly include povidone-iodine (PVI) as the antiseptic agent of choice for the preparation of ocular and periocular surfaces [2]. IC cefuroxime toward the end of the procedure is also a well-known standard for prevention in the international literature [7]. A local survey done in 2020 assessed nationwide practices in preventing post-op IE, and the results were far from ideal [8]. Only 66.3% of practitioners were using PVI, while 40.2% were practicing IC cefuroxime despite strong published evidence in favor of both. In our setup, however, both established protocols were practiced regularly. Oral ciprofloxacin, a second-generation fluoroquinolone, is also being prescribed as an adjunct for three to five days post-op.

The intraocular penetration of some broad-spectrum antibiotics, e.g., fluoroquinolones such as moxifloxacin and ciprofloxacin, is well-documented in the literature, but the most serious consequence of their routine prophylactic use is the potential threat of more virulent drug-resistant bacterial strains [9,10]. The culmination of a multitude of studies investigating molecular and environmental mechanisms of antimicrobial resistance is the restricted and judicious use of these medications, which are easily available over the counter and prescribed inappropriately without proven efficacy in most developing countries [11-13]. The role of oral antibiotics in preventing cataract surgery-related IE, even though routinely prescribed in most high-volume setups across Pakistan, is still controversial and not well-established in the literature.

It is of paramount importance for cataract surgeons to adopt the most effective and proven measures to prevent post-op IE. The purpose of our study is to establish the role of perioperative oral antibiotics in reducing the risk of IE after uncomplicated cataract surgery. This will provide us with a better understanding of the most effective prophylactic measures against this vision-threatening complication.

## Materials and methods

We conducted a prospective cohort study of all the patients >18 years who underwent uncomplicated phacoemulsification with intraocular lens implantation between August 1, 2022, and January 31, 2023, in the General Ophthalmology Department of Al-Shifa Trust Eye Hospital, Rawalpindi. The approval of the Institutional Review Committee (Ref No. ERC-16/AST-22) of Al-Shifa Trust Eye Hospital was obtained. This study adhered to the tenets of the Declaration of Helsinki.

Inclusion criteria 

All patients above the age of 18 years who underwent uncomplicated phacoemulsification during our study period were included. Patients with mature and immature senile cataracts were included.

Exclusion criteria

Patients with severe mental illness and developmental disabilities were excluded from the study because of concerns about inadequate post-op ocular hygiene and medication compliance. Eyes with coexisting blepharitis, dacryocystitis, and active ocular inflammation were strictly excluded from the surgical list. Post-traumatic eyes, complicated cataracts, and patients with intraoperative complications like posterior capsular rent or vitreous loss were also excluded. 

Noted data variables include age, gender, laterality, cataract density, surgeon level, and systemic use of antibiotics. The level I surgeon was a senior resident; level II was a registrar (a young ophthalmologist who has completed residency and/or fellowship), while level III surgeon was a consultant. Patients were followed for six weeks, and thorough slit-lamp examinations for anterior chamber (AC) reactions on post-op day 1, day 14, and day 45 were carried out. AC activity was graded using the conventional Standardization of Uveitis Nomenclature (SUN) grading system [14]. Acute post-op IE was defined as an inflammation of the inner coats of the eye resulting from intraocular colonization of infectious agents within six weeks of surgery [15].

Surgical technique

Multiple surgeons performed phacoemulsification using CataRhex 3®, Oertli Instruments Inc., Switzerland. The surgical steps were nearly consistent with either topical or peribulbar anesthesia, depending on the patient’s cooperation or preference. In all cases, clear corneal incisions of 2.75/3.2 mm with divide-and-conquer, stop-and-chop, or direct chop techniques were used at the discretion of the surgeon. Foldable in-the-bag intraocular lenses (RayOne Aspheric, Rayner, United Kingdom) were implanted and wounds were hydrated. There were minor variations in technique and expertise among surgeons. All surgeons had performed at least 200-250 phacoemulsifications prior to this study and the surgical duration varied from 8 to 20 minutes. 

Endophthalmitis prophylaxis

Patients were randomly divided into two groups. Groups A and B were both similar in all perioperative endophthalmitis prophylaxis measures except for the administration of prophylactic oral antibiotics starting a day before surgery and continuing for two days post-op. Group A received post-op oral ciprofloxacin 500 mg BD for three days, whereas Group B did not. All patients were draped and prepared with a meticulous aseptic technique. Local skin disinfection with 10% PVI was performed, followed by instillation of 5% PVI in the conjunctiva cul-de-sac for three minutes. No topical antibiotics were administered preoperatively, and the balanced salt solution did not include antibiotics. At the end of the phacoemulsification procedure, an IC injection of 0.05 mL of undiluted moxifloxacin 0.5% (Vigamox®, Alcon Laboratories, Fort Worth, TX, USA) was given to all patients. 

Topical antibiotic prophylaxis was commenced six hours post-surgery with 1) two moxifloxacin per hour, 2) two 1% prednisolone acetate per hour, and 3) tobramycin-dexamethasone eye ointment OD at night, prescribed for the first week. At one week follow-up, the above medications were discontinued, and four hourly tobramycin-dexamethasone combination eyedrops were prescribed for another two weeks. 

All the above-mentioned antibiotics were used in our study since they are most frequently practiced in our institute and across Pakistan. Oral ciprofloxacin has good ocular penetration, and IC moxifloxacin is cost-effective and easily available in our region. 

Statistical analysis

Statistical Package for Social Sciences (SPSS) Version 21.0 was used for data analysis. A priori power analysis could not be conducted due to the lack of any previous similar studies based on oral antibiotic prophylaxis for cataract surgery. For quantitative data, mean and standard deviation were used (mean ± SD) whereas percentages were used for qualitative data. An independent sample t-test was used to compare AC reactions between the two groups. A p-value of <0.05 was taken as statistically significant.

## Results

A total of 2161 patients were included in our study after fulfilling the inclusion criteria. The mean age in our data was 59.3 ± 14.9 (mean ± SD) years with a majority of females (n = 1134, 52.5 %). Most of the operated patients had right eye involvement (n = 1158, 53.6%), whereas surgeries were predominantly done for visually significant immature cataracts (n = 1812, 83.9%) by a level III surgeon (n = 987, 45.7%). Demographics are given in Table 1.

**Table 1:** Demographic features Level I, senior resident; level II, registrar; level III, consultant

Variable	Frequency (n)	Percentage (%)
Gender		
Male	1027	47.5
Female	1134	52.5
Laterality		
Right	1158	53.6
Left	995	46
Cataract density		
Immature	1812	83.9
Mature	349	16.1
Surgeon level		
Level I	219	10.1
Level II	955	44.2
Level III	987	45.7

Out of 2161 patients, n = 859 (39.8%) were included in Group A (antibiotic group), while n = 1302 (60.2%) were in Group B (no antibiotic group). No significant difference in AC reactions between the two groups was noted on day 1 (p = 0.67). Our results show two cases of toxic anterior segment syndrome (TASS) from Group A in two patients who presented on days 10 and 13, respectively. Both were treated with intensive topical steroids (1 hourly prednisolone acetate) with close observation and responded very well. There were 111 patients lost to follow-up on day 14. Among the remaining patients (n = 2050), no significant difference in AC reactions between the two groups (Group A = 832, Group B = 1218) was noted on day 14 either (p = 0.03).

Among 2161 patients, we report one case of post-op IE (0.04%) from Group A (n = 302, 0.07%) who presented on day 13 with visual acuity of counting fingers, 0.2 mm hypopyon, pupillary membrane, and dense vitritis. The patient was immediately admitted and started on oral moxifloxacin 400 mg OD, topical prednisolone acetate, and moxifloxacin 2 hourly along with a cycloplegic. A same-day vitreous tap was performed, and conventional intravitreal antibiotics ceftazidime 2.25/0.10 mL and vancomycin 1 mg/0.10 mL were injected. The same antibiotics were repeated after 48 hours; however, due to declining vision (hand movements), the patient was referred to a vitreoretina surgeon for an early pars plana vitrectomy with a silicon oil tamponade. On day 45, 224 patients were lost to follow-up. No statistically significant difference (p = 0.1) was found in AC reactions between the two groups (Group A; n = 781, Group B; n = 1156) among the remaining 1937 patients.

## Discussion

Post-op IE is one of the worst potential complications following routine cataract surgery. Prevention practices vary considerably around the world, and no clear consensus exists to date regarding optimal strategies to reduce its incidence [15]. One of the reasons lies in the striking variations in its epidemiology across the globe, and all existing literature unequivocally emphasizes the importance of prophylactic measures in incidence reduction. This raises the critical question: which prophylactic regime should we adopt to attain this common goal?

Our study focuses on the role of perioperative oral antibiotics in the prevention of IE after routine uncomplicated phacoemulsification. Our results suggest no benefit of oral ciprofloxacin in the reduction of post-op IE rates. Similarly, a retrospective study in Japan found no evidence in favor of perioperative systemic cefdinir and levofloxacin administration [16]. Instead, it reported increased systemic side effects, including abdominal pain and diarrhea. A clinical trial by the Endophthalmitis Vitrectomy Study Group recommended the omission of systemic antibiotic treatment to reduce toxic effects, costs, and length of hospital stays in the prevention or treatment of postoperative endophthalmitis [17]. Thus, oral antibiotics not only have no benefit in reducing IE but, in fact, may have a negative impact. 

Post-op IE affects the anterior as well as posterior segments of the eye, usually secondary to an exogenous infectious organism that gains ocular access during surgery. For the anterior segment, topical antibiotic penetration in the aqueous humor is sufficient, while for the vitreous humor, topical and systemic routes do not achieve desirable concentrations except for linezolid and fluoroquinolones, which have excellent vitreous penetration [18,19].

A local study from Agha Khan Hospital regarding microbiologic profiles of post-op endophthalmitis reported that among gram-positive bacteria, coagulase-negative Staphylococcus was the principal isolate (17%), and among gram-negative bacteria, it was Pseudomonas species (18.8%) [20]. Systemic levofloxacin and moxifloxacin have superior coverage and high intraocular bioavailability in the uninflamed eye [9,10]. Ciprofloxacin in our set-up has good coverage of *Pseudomonas aeruginosa*, but its coverage against coagulase-negative staphylococci is doubtful. Even with high intraocular penetration and suitable Pseudomonas coverage, our results do not support its benefit as a systemic prophylactic agent against post-op IE. 

To date, a wide range of guidelines have been established for prophylaxis against post-op IE at different times. A few commonly practiced guidelines are topical and systemic antibiotics, irrigation with PVI solution, injection of local antibiotics, and antibiotics in irrigating solution [21]. Annual clinical audits are regularly conducted in worldwide setups for review and revision of protocols. Unfortunately, in our country, practice protocols to prevent IE are not well defined nationally with wide variations in practice in both public and private sectors. A local survey conducted by the British Pakistani Ophthalmic Society (BPOS) in 2020 highlighted some alarming facts [8]. According to the survey, only 53.8% of participating surgeons from our country provided some sort of routine antibiotic prophylaxis with the use of PVI on the skin, with the conjunctival sac being the most popular (66.3%). We have conducted this study to ensure uniformity of practice for prophylaxis against post-op IE to include three major protocols: pre-op PVI antisepsis, IC moxifloxacin toward the conclusion of surgery, and post-op topical moxifloxacin for four weeks. 

PVI is the single most important preventive measure, especially in poor lid-hygiene conditions and low socioeconomic populations because of its low cost and rapid bactericidal action without the promotion of antimicrobial resistance and because of strongly supportive data [22,23]. Our results also favor the use of PVI antisepsis comprising lid skin disinfection with 10% PVI and a diluted 5% solution for instillation in the conjunctival sac, as previously described [24].

The European Society of Cataract and Refractive Surgeons reported a substantial fivefold reduction in a randomized clinical trial (2007) in IE rates associated with the use of IC cefuroxime [7]. Several studies have reported the safety of 1 mg in 0.1 mL of cefuroxime for intraocular administration [25,26]. Another retrospective analysis of about 600,000 eyes demonstrated a colossal sixfold reduction in IE rates after phacoemulsification with the use of IC moxifloxacin [27]. Our study also supports the value of IC moxifloxacin as a suitable prophylactic agent with an endophthalmitis rate of 0.046% in our subjects. Surveys show that nearly all surgeons prescribe prophylactic topical antibiotics, with most surgeons favoring the latest generation of fluoroquinolones, but no proof supporting their contribution has been reported [28,29]. We also prescribed topical moxifloxacin for all our subjects for at least one month post-operatively, supporting its use in IE prophylaxis. 

Our study provides level II evidence in favor of discarding the routine use of post-op oral ciprofloxacin in uncomplicated cataract surgeries. The strength of our study lies in the prospective and clear comparative nature of its design. Also, the inclusion of a uniform surgical technique and meticulous sampling of patients with a stringent application of our inclusion and exclusion criteria. It is generalizable because of the multiethnic patient population to which our charity hospital caters. Limitations of our study lie in the limited sample size, the inclusion of uncomplicated surgeries only and of surgeries by various levels of surgeons as training doctors with less experience, posing a higher risk of complications such as endophthalmitis [30]. Nationwide, multicenter studies and regular clinical audits involving both urban and rural areas are needed to assess the risk factors and incidence rates of endophthalmitis. Microbiologic profiles and antibiotic susceptibility studies should also be considered along with our findings to ascertain the most common culprits. We also suggest the importance of forming an endophthalmitis task force to formulate standard national practice guidelines for the prevention of endophthalmitis outbreaks.

## Conclusions

This prospective study analyzed the real-world data of consecutive cataract surgeries in a single high-volume surgical institution performed by multiple surgeons and showed that there is no significant difference in the rate of post-op IE, irrespective of the use of systemic antibiotics. Our study showed no benefit of using post-op oral ciprofloxacin in the presence of other prophylactic measures like preoperative conjunctival 5% PVI for five minutes and IC moxifloxacin at the end of surgery. Furthermore, studies with more power and different kinds of antibiotics are needed to establish stronger evidence for or against the use of oral antibiotics as a prophylactic measure for cataract surgery in routine practice.
